# Virological Profile of Asthma Exacerbation in Children: A Hospital-Based Retrospective Study

**DOI:** 10.7759/cureus.60261

**Published:** 2024-05-14

**Authors:** Basma Ennadif, Fatima Zahra Alaoui-Inboui, AbdelHakim Youssef Benmoussa, Assiya El Kettani, Naima Elmdaghri, Bouchra Slaoui

**Affiliations:** 1 Department of Pediatrics, Faculté de Médecine et de Pharmacie, Université Hassan II, Casablanca, MAR; 2 Pediatric Pneumo-Allergology Unit, Pediatric Department 2, Hôpital Mère-Enfants Abderrahim Harouchi, Centre Hospitalier Universitaire Ibn Rochd, Casablanca, MAR; 3 Department of Microbiology, Faculté de Médecine et de Pharmacie, Université Hassan II, Casablanca, MAR; 4 Department of Microbiology, Centre Hospitalier Universitaire Ibn Rochd, Casablanca, MAR

**Keywords:** respiratory syncytial virus, coinfections, rhinovirus, asthma exacerbation, virus

## Abstract

Introduction

Viruses are the most common triggering factors for asthma exacerbation during the autumn and winter seasons. Viruses, such as influenza A and rhinovirus, play a major role in the occurrence of severe exacerbation of asthma. This association between viral infection and asthma exacerbation in children is a result of the antiviral response of the immune system and various anti-inflammatory phenomena. In this work, we aimed to identify the virological profile of asthma exacerbation in children and analyze the correlation between viral infection type and the severity of exacerbation.

Materials and methods

This retrospective study was conducted from January 2016 to January 2024. The study included children hospitalized for asthma exacerbation associated with signs of viral-like respiratory infection with positive virological testing by multiplex real-time polymerase chain reaction or rapid test in the case of influenza A or respiratory syncytial virus (RSV). Data analysis was performed with Microsoft Excel and SPSS software using a previously established data collection sheet

Results

Thirty cases were collected for the study period. The mean age of the patients was 4 years and 8 months, with a male-to-female ratio of 3.3. Eighteen patients were known to have asthma, of which nine had uncontrolled asthma, and exacerbation was inaugural in 12 patients. Viral shedding was found in 14 patients. A viral agent was found in all patients, with coinfection of two or more viruses in three patients. The viruses found were influenza A (18 cases), coupled rhinovirus/enterovirus (eight cases), RSV (eight cases), human metapneumovirus (three patients), and parainfluenza type IV in only one inaugural patient. Asthma exacerbation was severe in 20 patients, moderate in eight patients, and two patients had severe acute asthma requiring intensive care management. We noted a higher frequency of severe exacerbation among those with an influenza A viral infection. All patients with RSV infection exhibited moderate exacerbation. No other significant correlation between asthma severity and other types of viruses was found.

Conclusions

Our results demonstrate the major role played by viruses in triggering asthma exacerbation, primarily influenza virus, followed by enterovirus, rhinovirus, RSV, and metapneumovirus. Larger-scale studies should be carried out to establish a more complete virological profile and further investigate the viral factor in the management of asthma in children.

## Introduction

Asthma is the most common chronic disease among children [1]. In 2019, the World Health Organization estimated that 262 million people suffered from asthma, resulting in 455,000 deaths. Severe exacerbation and associated hospitalization are the main contributors to the morbidity and financial burden of the disease [2]. Respiratory pathogens are the most common triggering factors for asthma exacerbation during the autumn and winter seasons, primarily among individuals with often uncontrolled asthma. Studies show that viral infection is frequently identified in the upper tract, as seen in up to 80% of pediatric cases [3]. Human rhinovirus/enterovirus is a major pathogen responsible for asthma exacerbation in children, as is respiratory syncytial virus (RSV), influenza virus, and human metapneumovirus (HMPV) [4]. RSV is most prevalent in early childhood, whereas human rhinovirus/enterovirus is more frequently detected in school-age children. Despite significant progress in prevention and management, asthma remains a substantial burden on global healthcare systems [5].

The objectives of this study were to identify the virological profile of hospitalized asthma exacerbation patients, and analyze the correlation between the type of virus identified and the severity of the exacerbation.

## Materials and methods

We conducted a retrospective study over an eight-year period from January 2016 to January 2024. The study utilized medical-record data of patients hospitalized for asthma exacerbation associated with symptoms suggestive of viral respiratory infection. We included those one month to 15 years of age with positive virological findings and excluded cases positive for SARS-CoV-2. The following signs were considered highly indicative of viral infection: runny nose, fever, fatigue, and digestive symptoms. Anamnestic, clinical, biological, and radiological data was collected for each patient. Anamnesis was used to determine whether the child was already known to have asthma or the asthma exacerbation was inaugural. Information about viral exposure and the presence of viral infection signs in the child was collected.

Physical examination provided information about the severity of signs of asthma exacerbation. Severe exacerbation was defined as an oxygen saturation (SaO_2_) below 90% and the presence of one or more of the following clinical signs: cyanosis, signs of intense respiratory distress, tachypnea, and faintly audible wheezing. Moderate exacerbation was defined as moderate dyspnea, clearly audible wheezing, and an SaO_2_ of 91%-94%. Bronchial secretion samples were analyzed using real-time polymerase chain reaction to detect and identify viruses or by rapid testing for influenza A, B, and RSV. A correlation study between clinical presentation and the detected virus was conducted using SPSS software (IBM Corp. Released 2021. IBM SPSS Statistics for Windows, Version 28.0.1.1 Armonk, NY: IBM Corp).

## Results

The study included 30 children aged 2-13 years, with a mean age of 4 years and 8 months. The male-to-female ratio was 3.3, with a male predominance. Eighteen patients were known to have asthma, among which nine had uncontrolled asthma, whereas exacerbation was inaugural in 12 patients. Viral exposure was found in 14 patients. The reported functional symptoms included a runny nose in 24 patients, dry cough in nine patients, and a productive cough in 13 patients, with digestive symptoms (e.g., diarrhea) reported in seven cases. Clinical signs found on physical examination included cyanosis in eight patients, tachypnea in 12 patients, and signs of respiratory distress in 24 patients. The SaO_2_ ranged from 88% to 94%.

Chest X-rays revealed alveolar consolidation in 16 patients, bronchial syndrome in 12 patients, and interstitial syndrome in two patients. C-reactive protein was positive in 12 patients, with an average value of 38 mg/dL. Neutrophil-predominant leukocytosis was found in 10 patients, lymphocytosis in six patients, and lymphopenia (<1000/mm^3^) in two patients. The viruses identified were influenza A in 18 patients, human rhinovirus/enterovirus in eight patients, RSV in eight patients, HMPV in three patients, and parainfluenza type IV in one patient (Table 1).

**Table 1:** Viruses identified in the study population.

Virus identified	Number of cases n (%)
Influenza A H1N1	18 (60%)
Rhinovirus/Enterovirus	8 (26.6%)
Respiratory syncytial virus	8 (26.6%)
Human metapneumovirus	3 (10%)
Parainfluenza	1 (3.3%)

Twenty patients experienced severe exacerbation, eight patients experienced moderate exacerbation, and two patients had severe acute asthma requiring intensive care management. All children with RSV infection had moderate exacerbation. Among the 18 cases with Influenza A H1N1 infection, six had severe exacerbation, and 12 had moderate exacerbation.

Viral co-infection was found in five patients: H1N1 and human rhinovirus/enterovirus coinfection occurred in two patients, RSV and human rhinovirus/enterovirus coinfection occurred in two patients, and triple viral coinfection involving HMPV, RSV, and human rhinovirus/enterovirus was observed in one patient (Table 2).

**Table 2:** Viral coinfection among the study population.

Coinfection type	Number of cases (n)
Influenza A H1N1 + Rhinovirus/Enterovirus	2
Respiratory Syncytial Virus + Rhinovirus/Enterovirus	2
Metapneumovirus + Respiratory Syncytial Virus + Rhinovirus/Enterovirus	1

Among our small study population, we did not find any significant correlation between the type of respiratory virus and the severity of exacerbation.

## Discussion

The link between viral infection and asthma exacerbation is well established. In one study, viral infection was responsible for triggering asthma exacerbation in 80% of child cases and over 50% of adult cases [6]. Several studies have reported a predominance of rhinovirus infections, as seen in Table 3 [1,2,7-11].

**Table 3:** Comparison of the percentage of viruses triggering asthma exacerbations

Study	Brouard et al. [1]	Thumerelle et al. [2]	Khetsuriani et al. [7]	João Silva et al. [8]	Maffey et al. [9]	Prazma et al. [10]	Abe et al. [11]	This Study
Study period	2 years	1 year	1 year	1 year	1 year	4 months	3 years	8 years
Year of the study	2001	2003	2006	2007	2010	2015	2019	2024
Location	Caen, France	Nord-Pas de Calais region, France	Emory, Georgia	Oporto, Portugal	Buenos Aires, Argentina	USA (39 states)	Yamaguchi, Japan	Casablanca, Morocco
Number of cases	180	82	65	54	209	339	216	30
Percentage of patients with isolated virus	77%	38%	63%	78%	78%	64%	75%	-
Rhinovirus	45%	12%	60%	46%	24%	80%	48%	28%
Respiratory syncytial virus	28%	7%	1.5%	27%	40%	-	6%	25%
Human metapneumovirus	-	-	4%	-	-	-	4%	10%
Influenza A	5.5%	-	-	-	-	4%	4%	64%

Viruses, including RSV, coronaviruses, parainfluenza and influenza viruses, enteroviruses, HMPV, and the newly described bocavirus, were found either alone or in co-infection during asthma exacerbation [6]. There is no significant difference in the severity of asthma exacerbation between those with single-infection and co-infection groups (i.e., viral or bacterial), suggesting that superinfection does not exert additional effects on asthma exacerbation [3,11]. Atypical bacteria still rank second in exacerbation, especially *Mycoplasma pneumoniae,* followed by *Chlamydia pneumoniae* [12].

In our study, influenza A H1N1 infection was the predominant virus type (64% of cases), followed by human rhinovirus/enterovirus and RSV. The studies listed in Table 3 included all children with asthma exacerbation of any severity, treated in-hospital or as outpatients, whereas our study included only patients with moderate or severe asthma exacerbation with signs of viral infection, treated in-hospital. A literature review published in 2018 examined the prevalence of viruses during asthma exacerbation across 63 studies, including 29 cross-sectional studies, 13 case-control studies, 14 cohort studies, and seven prospective studies, among which prevalence varied considerably [5]. This heterogeneity of prevalence was most likely due to differences in study design, ethnic origin, geographical region, site of sample collection, and detection methods. The most frequently found virus is rhinovirus (42%), followed by RSV (13%) and influenza (10%) [12].

A study conducted in Seoul, South Korea, revealed variations in the age distribution of infants and children associated with different viral infections [13]. Asthma exacerbation was predominantly caused by RSV among younger infants (mean age of 23 months), whereas rhinovirus or influenza viruses were detected in older children (mean ages of 41 months and 62 months, respectively). The study found no difference in triggering viruses among different age groups. Additionally, no difference in virus distribution was found based on gender, which is consistent with the findings of our study.

Several studies have discussed the mechanisms of susceptibility to rhinovirus infections in asthma. Innate and adaptive immune cells located in the respiratory mucosa play a crucial role in antiviral defenses and asthma inflammation. Viral infection of the respiratory tract typically induces an antiviral response associated with a Th1-type immune reaction. Such responses are also associated with the production of type I interferons (IFNs). In asthmatic patients, the antiviral response tends to be mainly Th2-type, which amplifies the antiviral inflammatory response and triggers exacerbation [14,15]. In their in vitro study, Wark et al. demonstrated a deficiency in innate immune response in bronchial epithelial cells among asthmatic patients; in response to rhinovirus infection, these cells showed defective IFN production compared to healthy bronchial epithelial cells, resulting in increased viral replication and cell lysis deficiency [16]. IFN production was found to be inversely correlated with bronchial inflammation, viral load, and the severity of symptoms.

The prevalence of specific viruses associated with asthma exacerbation varies considerably across geographic regions. Zheng et al. reported that rhinovirus was most prevalent in Asia, Europe, America, and Oceania, whereas RSV was most prevalent in Africa, possibly owing to its generally higher prevalence in low-income countries [5,17]. In vitro studies have demonstrated that exogenous administration of interferons (e.g., IFN-α, IFN-β, IFN-λ1, or IFN-λ2) reduces the viral load of rhinovirus 1A in primary bronchial epithelial cells [18]. Furthermore, the addition of IFN-β also suppresses the replication of RV16 and RV1B in primary bronchial epithelial cells isolated from both healthy individuals and asthmatic patients. A study by Djukanovic et al. evaluated the therapeutic effect of inhaled IFN-β on asthmatic patients at the onset of cold or flu symptoms, suggesting it is a potential treatment for virus-induced asthma exacerbation in difficult-to-treat patients [19]. More recently, in 2020, Watson et al. reported the effect of intermittent prophylactic doses of exogenous IFN-β to modulate viral infection in an in vitro model [20].

Limitations of the study

This study did have some limitations. First, as the study sample was small, our results need to be further verified in future works with larger samples of asthmatic children. Second, this was a hospital-based study, which could be supplemented with investigations into viruses triggering mild exacerbation commonly seen in outpatient settings.

## Conclusions

Viral infections are largely responsible for asthma exacerbation in children. In this small, hospital-based study, influenza A was the most frequently encountered virus, followed by enterovirus, rhinovirus, RSV, and HMPV. Our results underscore the value of flu vaccination in asthmatic children. However, a larger-scale study would be desirable to establish a more complete virological profile.
